# Allelic imbalance at the *HER2/TOP2A* locus in breast cancer

**DOI:** 10.1186/s13000-015-0289-x

**Published:** 2015-05-29

**Authors:** Cornelis J. J. Huijsmans, Adriaan J. C. van den Brule, Henny Rigter, Jeroen Poodt, Johannes C. van der Linden, Paul H. M. Savelkoul, Mirrian Hilbink, Mirjam H. A. Hermans

**Affiliations:** Laboratory of Molecular Diagnostics, Jeroen Bosch Hospital, PO Box 90153, 5200 ME ‘s-Hertogenbosch, The Netherlands; Laboratory of Pathology, Jeroen Bosch Hospital, PO Box 90153, 5223 GZ ‘s-Hertogenbosch, The Netherlands; Medical Microbiology and Infection Control, VU University Medical Center, PO Box 7057, 1007 MB Amsterdam, The Netherlands; Department Medical Microbiology, Maastricht University Medical Center, PO box 5800, 6202 AZ Maastricht, The Netherlands; Jeroen Bosch Academy, Jeroen Bosch Hospital, PO Box 90153, 5223 GZ ‘s-Hertogenbosch, The Netherlands

**Keywords:** Breast cancer, Allelic instability, *HER2*, *TOP2A*, Single nucleotide polymorphism

## Abstract

**Background:**

Breast cancer is a heterogeneous disease with various histological features and molecular markers. These are utilized for the prediction of clinical outcome and therapeutic decision making. In addition to well established markers such as HER2 overexpression and estrogen and progesterone receptor (ER and PR) status, chromosomal instability is evolving as an important hallmark of cancers. The *HER2/TOP2A* locus is of great importance in breast cancer. The copy number variability at this locus has been proposed to be a marker for the degree of chromosomal instability. We therefore developed a Single Nucleotide Polymorphism (SNP) assay to evaluate allelic imbalance at the *HER2/TOP2A* locus in three different entities of primary breast tumors.

**Methods:**

Eleven SNPs were carefully selected and detected by real time PCR using DNA extracted from paired (histologically normal and tumor) paraffin-embedded tissues. Primary breast tumors of 44 patients were included, 15 tumors with HER2 overexpression, 16 triple negative tumors, defined by the absence of HER2 overexpression and a negative ER and PR status and 13 ER and PR positive tumors without HER2 overexpression. As controls, histologically normal breast tissues from 10 patients with no breast tumor were included.

**Results:**

Allelic imbalance was observed in 13/15 (87 %) HER2 positive tumors, the remaining 2 being inconclusive. Of the 16 triple negative tumors, 12 (75 %) displayed instability, 3 (19 %) displayed no instability, and 1 was inconclusive. Of the 13 hormone receptor positive tumors, 5 (38 %) displayed allelic imbalance, while 8 did not.

**Conclusions:**

We conclude that the SNP assay is suitable for rapid testing of allelic (im)balance at the *HER2/TOP2A* locus using paraffin-embedded tissues. Based on allelic imbalance at this locus, both triple negative and ER and PR positive breast tumors can be subcategorized. The clinical relevance of the allelic (im)balance status at the *HER2/TOP2A* locus in breast cancer is subject of future study.

**Virtual Slides:**

The virtual slide(s) for this article can be found here: http://www.diagnosticpathology.diagnomx.eu/vs/2086062232155220

**Electronic supplementary material:**

The online version of this article (doi:10.1186/s13000-015-0289-x) contains supplementary material, which is available to authorized users.

## Background

Breast cancer is a heterogeneous disease that can display a diverse variety of biological features. Several proteins are well known to be expressed excessively in these tumors. Based on the excessive presence of such proteins, breast cancer can be divided in several different biological entities. This breast cancer categorization is utilized for the prediction of clinical outcome and therapy selection [1].

Well known examples of overexpressed proteins that play a critical role in breast cancer diagnostics and choice of therapy, are the estrogen and progesterone receptors (ER and PR). Available targeted therapies in these breast cancer cases are e.g. Tamoxifen and aromatase inhibitors [2–4].

In addition to the overexpression of ER and PR, aberrations of the chromosome 17q12-q22 genomic region play an important role in breast cancer. This locus incorporates the *HER2* gene as well as the juxtaposed *TOP2A* gene, which is located 700 kb telomeric of *HER2* [5–7]. The *HER2* oncogene encodes the human epidermal growth factor receptor 2 protein, a 185 kDa transmembrane tyrosine kinase receptor also known as erbB-2 and *neu* [5, 6]. Upon ligand binding the other types of HER receptors will dimerize with HER2. Such dimerization triggers an integrate network of HER signal transduction involving a variety of signaling pathways [8–10]. When the *HER2* oncogene is amplified, *HER2* overexpression occurs resulting in overstimulation of HER mediated signal transduction [8]. *HER2* is found to be amplified in 25–30 % of breast cancers [11]. Patients with *HER2* amplified breast tumors resulting in overexpression of HER2, benefit from treatment with the monoclonal antibody Trastuzamab (Herceptin). Trastuzamab is thought to abolish the HER2 mediated signaling of the HER signal transduction network [12, 13]. In addition, analysis of several studies has shown an association of improved adjuvant anthracycline (AC)-based chemotherapy response rates in *HER2* amplified breast tumors [14]. However, there is no clear AC induced HER2 inhibiting mechanism, it is therefore suggested that *TOP2A* coamplification is the biological rationale behind these observations [15]. The *TOP2A* gene encodes topoisomerase II alpha, a 170 kDa enzyme that catalyzes the topological DNA changes needed during the multistep process of cell division [16]. Coamplification of *TOP2A* occurs along with *HER2* amplification in approximately 30–45 % of the *HER2* amplified cases [15, 17, 18]. Patients with *TOP2A* amplified breast tumors seem to benefit from adjuvant AC-based chemotherapy. ACs are chemotherapeutic agents that are known to have, among other mechanisms of action, TOP2A inhibiting properties. TOP2A catalytic activity is inhibited by ACs via stabilization of the intermediate complex where TOP2A tyrosine residues are covalently linked to the 5’ phosphoryl ends of the cut DNA strands during the topological changes which they catalyze. Trapping the complex in this state prevents DNA resealing and further cell cycle progress, which ultimately induces programmed cell death [16, 19, 20]. In patients that are treated with AC-based chemotherapy, breast tumors with *TOP2A* amplification show a trend towards a better disease free survival than tumors lacking this amplification. In addition, *TOP2A* amplification is an independent predictor of a significantly higher pathological complete response rate in these patients [18]. On the contrary, *TOP2A* deletion, occurring in 15–40 % of *HER2* amplified cases, has been associated with AC resistance [15, 17, 18]. Tumors lacking ER and PR overexpression as well as HER2 amplification are defined as “triple negative” breast tumors. Triple negative tumors are associated with poor clinical outcome and lack of (currently) available targeted therapy [21, 22].

The above mentioned biological characteristics are well established biomarkers utilized to categorize breast cancer types for diagnostic and treatment related purposes. More recently, high resolution array based systems, targeting Single Nucleotide Polymorphisms (SNPs) or utilizing Comparative Genomic Hybridization, have been employed to subcategorize these breast tumor types and revealed distinct profiles of gene-expression or genetic alterations [23–28]. Hormone receptor positive tumors without *HER2* overexpression showed minor and less frequent chromosomal instability in comparison to *HER2* overexpressed and triple negative tumors that showed more extensive and more frequent chromosomal instability [23–29]. Copy number alterations across the genomic region incorporating the *HER2/TOP2A* locus have been proposed to be a marker for the degree of chromosomal instability in breast cancer [30, 31]. Early breast cancer patients with a tumor containing extensive chromosomal instability, that were treated with AC-based therapy, were shown to have a decreased risk of death and a trend towards a decreased risk of relapse. Similarly treated patients with a tumor of low chromosomal instability showed no apparent benefit [30, 31]. Determining such instability in a diagnostic setting may therefore be of clinical relevance as an adjuvant molecular marker.

Single Nucleotide Polymorphisms (SNP) are easily detected by real time PCR, a technique that is widely available in many laboratories that routinely employ molecular diagnostics. By analyzing a panel of heterozygous SNPs, copy number alterations and thereby allelic imbalance of the involved locus can be determined. It therefore may provide an accessible method to assess allelic imbalance at the *HER2/TOP2A* locus.

## Methods

### Study population and clinical specimens

The study was carried out retrospectively with the approval of the Board of Directors and the Scientific Advisory Board of the Jeroen Bosch Hospital. All patient samples were rendered anonymous before use in this study.

#### Control group

Ten histologically normal formalin-fixed paraffin-embedded (FFPE) breast -tissues from 10 different patients (CT1-10) with no breast tumor were included as controls (Additional file 1: Table S1). Age of included patients was 32.8 ± 12.0 years (mean age ± 1 standard deviation (STD)).

#### Breast tumor patient group

Breast tumors (FFPE tissue) with known *HER2* immunohistochemistry (IHC) and silver *in situ* hybridization (SISH) status from 44 patients were included in this study (Additional file 2: Table S2): 15 tissues with *HER2* amplification (IHC 3+, patients HER2 + 1–15; aged 52.9 ± 14.2 years (mean age ± 1 STD)) 16 triple negatives (patients TN1-16; aged 53.4 ± 12.9 years (mean age ± 1 STD)) and 13 ER/PR positive and *HER2* non-amplified (ERPR+) breast tumors (patients ERPR+ 1–13; aged 55.5 ± 9.3 years (mean age ± 1 STD)). To establish the germline SNP profile, a histologically normal archival FFPE tissue (breast tissue or lymph node tissue in case no breast tissue was available; Additional file 2: Table S2) from each patient was analyzed.

### Tissue Processing

Tissues were fixed and processed as previously described [32]. Three-μM-thick sections were cut from each FFPE tissue and mounted on glass slides in sevenfold. One was used for Hematoxylin & Eosin (HE) staining, two to four (depending on tissue size, with a total of approximately 2 cm^2^) for genomic DNA extraction, one for SISH and one for IHC. The latter two were only performed for the tumor samples.

### HER2 immunohistochemistry

To establish the HER2 expression status, IHC was performed on all 44 tumor samples using the Ventana anti-Her2/neu(4B5) (Roche Diagnostics GmbH, Mannheim, Germany) in combination with the VENTANA system (Roche Diagnostics GmbH) according to the manufacturers protocol. IHC 0 and 1+ were considered to represent normal expression, 2+ was considered borderline with regard to expression and 3+ was considered to represent overexpression.

### *HER2* silver in situ hybridization

To determine the *HER2* amplification status and to assess chromosome 17 copy number status, SISH was performed on all 44 tumor samples using the INFORM HER2 Dual colour SISH DNA probe cocktail (Roche Diagnostics GmbH) in combination with the VENTANA system (Roche Diagnostics GmbH) according to the manufacturers protocol. Twenty nuclei were counted independently by 2 individuals. The mean from these 2 nuclei counts was calculated. Interpretation was as follows: a ratio (*HER2* probe/centromere probe) of <1.8 represented no amplification; a ratio of 1.8–2.2 was equivocal and a ratio of >2.2 represented amplification.

### Genomic DNA extraction from FFPE tissues

From each FFPE tissue, tumor as well as (reference) histologically normal tissue, a HE-slide was made. The tumor tissue containing HE slides were used to determine the area with the highest tumor cell percentage (TC%). The HE slides containing the histologically normal tissue were used to determine the location of the highest nuclei density. Subsequently, these were used as a reference for tumor and histologically normal cell macrodissection, respectively. The respective cells were scraped off the corresponding blank slides. The scrapings were used for digestion and subsequent EasyMAG NucliSens (BioMérieux Benelux BV, Zaltbommel, The Netherlands) DNA extraction as described previously [32].

### SNP selection

From the TaqMan® SNP Genotyping Assays from Applied Biosystems, 11 suitable SNPs were chosen using SNP browser software Version 4.0 (Applied Biosystems, Table 1). SNP 1–6 were located up- and downstream of the *HER2* gene within the *HER2* smallest region of amplification [33]. The remaining 5 SNPs (7–11) were located up- and downstream of the *TOP2A* gene. The selected SNPs displayed an allele frequency of approximately 0.5 in Caucasian (CEU, CEPH (Centre d'Etude du Polymorphisme Humain) from Utah), Chinese (CHB, Chinese from Beijing), Japanese (JPT, Japanese from Tokyo) and African (YRI, Yoruba from Ibadan Nigeria) populations. The latter to maximize the chance for heterozygosity, independent of the patients’ ethnicity, for only those SNPs are informative.

**Table 1:** Panel of selected Single Nucleotide Polymorphisms (SNPs)

SNP nr	dbSNP-ID (rs)	SNP-ID (hCV)	Base (17q)	SNP	Minor allele frequency			
					CEU	CHB	JPT	YRI
1	7214151	30187206	34,761,298	C/T	0.38	0.04	0.45	0.46
2	7503195	437172	34,976,041	A/G	0.38	0.04	0.45	0.45
3	876493	7452397	35,078,071	G/A	0.36	0.23	0.43	0.43
4	4795408	30547514	35,361,153	A/G	0.47	0.43	0.47	0.39
5	4794822	31651885	35,410,238	T/C	0.42	0.38	0.39	0.39
6	868150	471861	35,466,885	A/G	0.42	0.34	0.44	0.45
7	2015561	7479296	35,846,755	G/A	0.46	0.44	0.42	0.00
8	4890114	27932396	35,857,075	T/C	0.46	0.44	0.42	0.00
9	8065040	11876205	36,073,767	A/G	0.37	0.43	0.43	0.36
10	10491123	29903401	36,086,722	A/G	0.37	0.44	0.44	0.47
11	7502428	29885409	36,133,740	C/T	0.47	0.44	0.43	0.41

SNP-ID (rs) = reference SNP ID number. SNP-ID (hCV) = Celera SNP ID. Base (17q) = the nucleotide position on chromosome 17(q). The *HER2* gene is positioned at base 35,097,919-35,138,441, the *TOP2A* gene at 35,798,321-35,827,695 (SNP browser software Version 4.0, Applied Biosystems). SNP 1-6 were located up- and downstream of the *HER2* gene within the *HER2* smallest region of amplification [33]. The remaining 5 SNPs (7-11) were located up- and downstream of the *TOP2A* gene. The particular nucleotide variation is referenced in “SNP”-column. Minor allele frequencies are indicated for different populations: CEU, CEPH (Centre d'Etude du Polymorphisme Humain) from Utah; CHB, Chinese from Beijing; JPT, Japanse from Tokyo and YRI, Yoruba from Ibadan Nigeria

### Allelic instability testing by SNP detection

For real time PCR based amplification and detection of the selected SNPs, corresponding predesigned TaqMan® SNP Genotyping Assays were purchased (Applied Biosystems, Foster City CA, USA; Table 1) containing two primers and two MGB TaqMan probes (5’ VIC for allele 1, 5’ FAM for allele 2 and a 3’ black hole quencher for both alleles). The SNP assays were used according to the manufacturer's instructions. Real time PCR based SNP detection was performed as previously described [32], in an ABI Prism 7500 FAST SDS (Applied Biosystems) machine for 1 min at 95 °C, followed by 45 cycles of 3 s at 95 °C and 30 s at 60 °C. To maximize pipetting accuracy and thereby minimize its influence on the data generated, all initial PCR setups were performed using the Hamilton StarLet instrument (Hamilton Robotics, Bonaduz, Switzerland).

**Fig. 1:**
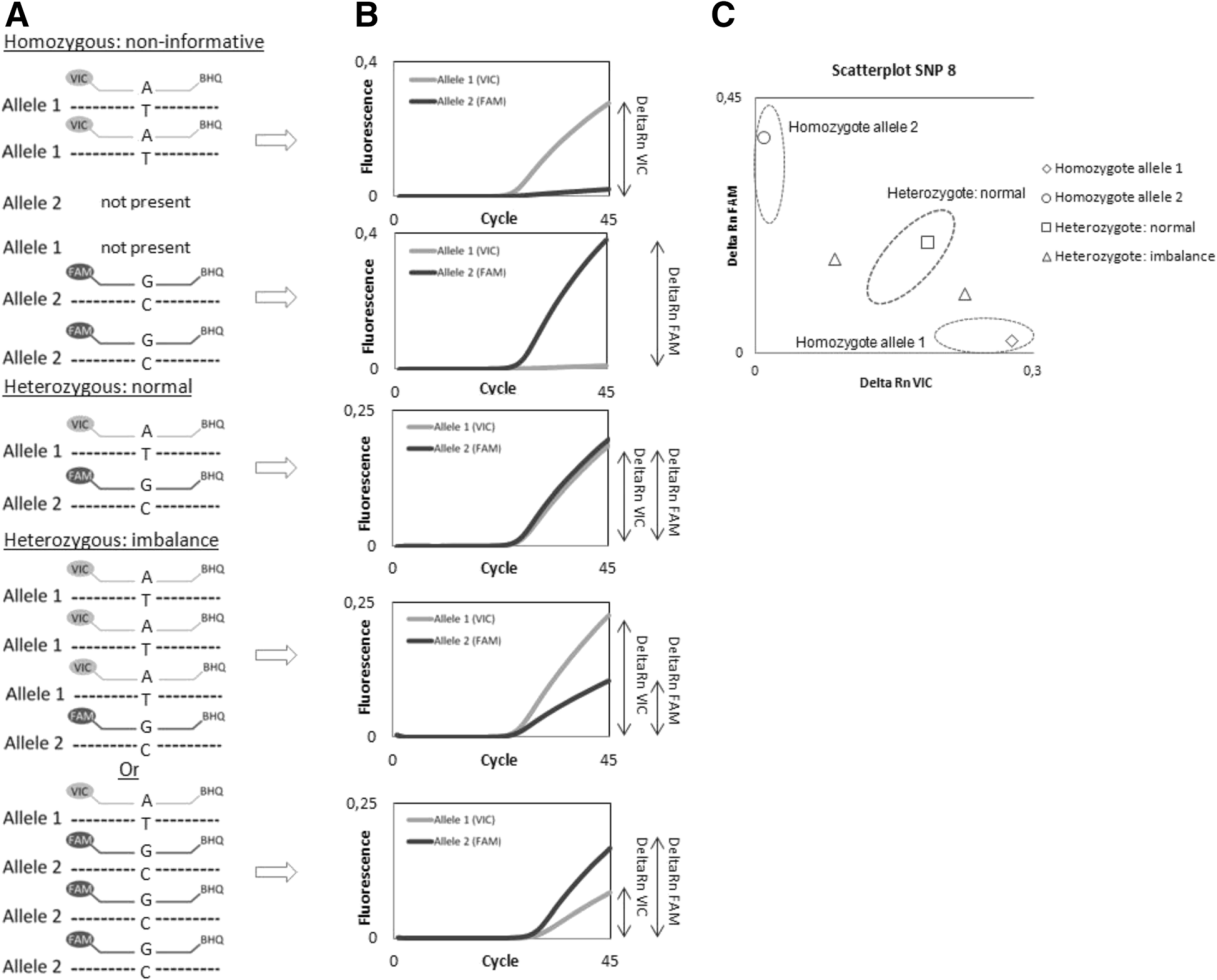
Schematic representation of allelic instability testing by real time PCR based SNP detection. **a.** Possible genotypes, from top to bottom: homozygous allele 1 and homozygous allele 2 (both non-informative), heterozygous normal and heterozygous imbalance (a 3:1 ratio of allele 1 vs. 2 and vice versa as a conceptual example). The probe corresponding to allele 1 is labeled with a 5’ VIC reporter, the allele 2 probe is labeled with a 5’ FAM reporter. Both probes contain a 3’ black hole quencher (BHQ). A = Adenine, T = Thymine, G = Guanine and C = Cytosine. **b.** The corresponding real time PCR component plots of above genotypes of representative SNP 8. X-axis: number of cycles, Y-axis: fluorescence. The arrows indicate the approximate delta Rn of the respective VIC or FAM reporters. **c.** Resulting scatterplot of SNP 8 of encountered genotypes. Dotted ovals indicate hypothetical homo- and heterozygous clusters. X-axis: delta Rn VIC, Y-axis: delta Rn FAM

Subsequent analysis was based on the generated fluorescence emission intensity of the VIC and FAM reporter dyes divided by the fluorescence emission intensity of the passive reference ROX dye (delta Rn value). When both alleles were present, a delta Rn VIC/FAM ratio could be established (allele 1/allele 2). This ratio was considered to be informative with regard to the relative number of alleles present and therefore a reflection of the allelic (im)balance status. Only the SNPs that were heterozygous for a given patient were informative. A schematic representation of this experimental scheme is presented in Fig. 1.

For each informative SNP the VIC/FAM ratios were plotted in a scatterplot. To minimize the influence of DNA concentration on VIC/FAM ratio and therefore data interpretation, the delta Rn FAM was corrected. The correction was performed by adapting the delta Rn FAM values of the histologically normal samples per SNP according to the crossing point of the trend line with the y-axis. This resulted in the translocation of the trend lines of the histologically normal tissues so that they all passed through the origin. All delta Rn FAM data of the tumor samples were subsequently corrected accordingly.

### Statistical analysis allelic instability testing

To gain insight into the distribution of delta Rn VIC/FAM ratios (ratio allele 1/allele 2), the 25^th^ and 75^th^ percentiles of the ratios generated by all histologically normal tissues (from the control group as well as the group of breast tumor patients) were calculated for each SNP assay using Microsoft Office Excel 2007 (Microsoft, Redmond WA, USA). The distance between these percentiles represents the interquartile range (IQR), which was used to establish cut-off values for the normal ratio distribution per SNP. The +1.5 IQR and −1.5 IQR were taken as cut-off value for normal ratio distribution for 10/11 SNPs (all SNPs except SNP 3). All ratios with an IQR of <1.5 follow normal distribution and were interpreted as genetically stable with regard to the *HER2/TOP2A* locus. Ratios plotted within 1.5–3 x IQR were considered equivocal with regard to *HER2/TOP2A* allelic instability status. Ratios plotted ≥3x IQR were considered to be representative for *HER2/TOP2A* allelic instability. Due to the large IQR generated using SNP 3 (varying from 2.3 to 15.1 times as large as found with the other SNPs), the cut-off values for that particular SNP were defined as follows: normal = −1 to +1 IQR; equivocal = 1–2 IQR and ≥2 IQR = indicative of allelic instability.

When a sample generated an equivocal result for any of the employed SNPs, the analysis of that particular sample was repeated in duplicate (with non-diluted DNA and with 5-fold diluted DNA in Tris Low EDTA buffer) using the respective SNP. When 2/3 results corresponded, the sample was called accordingly (e.g. equivocal/equivocal/normal was called equivocal). When results were conflicting, the sample was called equivocal (e.g. equivocal/allelic instability/normal).

## Results

Two patient groups were used in this study: a control group of 10 histologically normal tissues from patients (CT1-10) with no breast tumors and a group of 44 breast tumor patients with different entities of primary breast cancer (HER2 + 1–15, TN1-16 and ERPR+ 1–13) of which both tumor and normal tissues were available.

### SNP selection

Technical validation of the selected SNP assays was performed using the 10 histologically normal breast tissues from the control group. All SNP assays had a sufficient sensitivity, yielding cycle-threshold values of <35 (threshold 0.007), a cut-off above which results are routinely considered less reliable in our laboratory. In addition, little or no cross-reaction of the SNP assays’ allele 1 corresponding probes (VIC reporter) with allele 2, and of the allele 2 corresponding probes (FAM) with allele 1 was observed. This resulted in clear clustering of homozygous allele 1, homozygous allele 2 and heterozygous patients. The panel of 11 SNP markers (Table 1) was therefore used to analyze the clinical specimens (Additional file 1: Table S1 and Additional file 2: Table S2).

### SNP analysis of patient materials

**Fig. 2:**
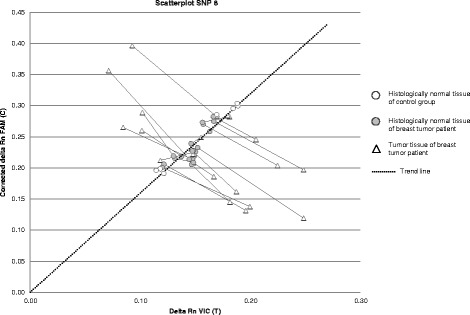
Visualization of VIC/FAM ratios that were generated using representative SNP 8 after FAM correction. Scatterplot of SNP 8 analyses of histologically normal tissues from the control group and paired histologically normal and tumor tissues from the group of breast tumor patients. Connected symbols indicate normal and tumor tissue of a single patient. X-axis: delta Rn VIC (T = Thymine), Y-axis: corrected delta Rn FAM (C = Cytosine)

To minimize the influence of DNA concentration on VIC/FAM ratio and therefore data interpretation, a correction on all delta Rn FAM data was performed as described in the Materials and Methods section. Fig. 2 shows the corrected scatterplot of representative SNP 8 for histologically normal tissues from the control group and paired histologically normal and tumor samples of all patients in the breast tumor group. Ratios of each sample pair (histologically normal and tumor tissues) were connected.

Subsequently, the delta Rn VIC/FAM ratios of all informative SNPs of the histologically normal tissues from both groups (10 tissues from the control groups and 44 tissues from the breast tumor group) were used to gain insight in normal data distribution. Combining the data of the histologically normal tissues from these groups was justified due to the mutual similarity of delta Rn VIC/FAM ratio distribution amongst both groups. To determine the *HER2/TOP2A* allelic (im)balance, fluorescent ratios of the panel of 11 informative SNP’s were analyzed using the cut-off values based on the interquartile range of data distribution amongst the histologically normal samples.

#### Histologically normal tissues

As expected, all informative SNP results (total of 54 SNPs of 10 patients) of the breast tissues from the control group fell within the normal IQR range (−1.5 to 1.5 IQR for SNP 1, 2 and 4–11 and −1 to 1 IQR for SNP 3). Overall results are depicted in Additional file 1: Table S1, Fig. 2 represents the corrected scatterplot of representative SNP 8.

The 44 histologically normal samples of the breast tumor patient group generated SNP results that fell within the normal IQR range and are therefore in agreement with the normal range observed in the control group. Only 1 out of the total of 208 informative SNPs yielded an equivocal result (patient TN2, SNP 11, triplicate analysis yielded 2 equivocal results and 1 result was found to indicate allelic instability).

#### Breast tumor tissues

Results of IHC and SISH are depicted in Additional file 2: Table S2. The SNP assays were used to investigate in more detail the genomic composition of the *HER2/TOP2A* locus. The results are summarized in Additional file 2: Table S2.

### HER2+ group

Within the group of patients with tumors overexpressing HER2, the SNP data of patients HER2+ 2–5 and 7–15 were consistently indicative of an instable *HER2/TOP2A* locus. IHC scores were 3+, SISH ratios were 2.0–15.2 and TC% ranged from 40 to 80 %. SNP 1 in patient HER2+ 8 generated an equivocal result. SNP results from patient HER2+ 1 and 6 showed inconsistent results between informative SNPs. All informative SNPs were therefore subsequently repeated in duplicate for these patients yielding identical results. SNP results from patient HER2+ 1 showed allelic balance of SNPs 8 and 9, whereas results from SNPs 1 and 10 were found to indicate allelic instability. IHC yielded a 3+ score, whilst the SISH ratio was 1.1. The sample had a TC% of 30 %. SNP results from patient HER2+ 6 were also inconsistent. SNPs 1, 2 and 8 were indicative of allelic stability, while results from SNPs 4, 7 and 11 showed allelic instability. IHC, SISH ratio and TC% in this patient were 3+, 4.1 and 80 %, respectively.

### Triple negative group

In the triple negative group, patients TN1-4, 6, 9, 10 and 12–16 showed SNP results consistent with instability of the *HER2/TOP2A* locus. IHC scores, SISH ratios and TC% varied from 0–2+, 0.7–1.9 and 25–90 %, respectively. Several SNPs generated equivocal results in patients TN1 (SNP 3 3x equivocal), 3 (SNP 4 3x equivocal), 8 (SNP 1 2x equivocal, 1x normal and SNP 2 3x equivocal), 10 (SNP 3 2x equivocal, 1x allelic instability) and 13 (SNP 1 and 3 both 3x equivocal). Patients TN5, 7 and 11 revealed no signs of *HER2/TOP2A* allelic imbalance, IHC scores were 0–1+, SISH yielded ratios of 1.1–1.6 and the samples contained 30–95 % tumor cells. In patient TN8, the 2 SNPs that were informative (SNPs 1 and 2) yielded equivocal results. IHC score, SISH ratio and TC% for this patient were 1+, 0.9 and 70 %, respectively.

### Estrogen and progesterone positive group

From the ERPR+ group, patients ERPR+ 1–3, 6 and 7 yielded SNP results indicative of *HER2/TOP2A* locus instability. IHC scores, SISH ratios and TC% varied from 0–1+, 0.5–1.1 and 30–70 %, respectively. Equivocal results were generated in patients ERPR+ 3 (SNP 3 3x equivocal) and 7 (SNP 3 1x equivocal, 1x allelic instability and 1x normal). The ratio of SNP 3 was found to be equivocal (equivocal, allelic instability and normal) in patient ERPR+ 7. The breast tumor tissues from patients ERPR+ 4, 5 and 8–13 showed a stable *HER2/TOP2A* locus, IHC scores were 0–1+, SISH ratios ranged from 1.0–1.5 and a TC% was 50–70 %. Patient ERPR+ 8 and 11 generated an equivocal result utilizing SNP 8 (2x equivocal, 1x allelic instability) and 4 (3x equivocal), respectively.

## Discussion

We have shown that the presented SNP based real time PCR assay is easy to perform and can be employed to reveal allelic instability of the *HER2/TOP2A* locus in breast tissues. The assay yielded results that fell within the normal range of data distribution in the control tissues. SNP results were consistent (all informative SNPs stable or instable) within most (42/44) tumor samples and were indicative of abundant allelic imbalance in *HER2* amplified and TN tissues.

In all patients (15/15) where HER2 overexpression was observed by IHC, SNP results were found to be indicative of *HER2/TOP2A* allelic instability. In 2 patients conflicting results between informative SNPs were found. In one sample (patient HER2+ 1), SNP 1 (instable in 2/3 observations and 1 equivocal) and SNP 10 results showed allelic instability while no allelic instability was observed for SNP 8 and 9. SISH analysis of this sample revealed no genomic amplification of the *HER2* locus. This possibly implies a false positive IHC result or *HER2* overexpession not related to gene amplification, which has been reported in 3–8 % of breast tumors [34–36]. However, the SNP assay did show the presence of a genetic aberration of part of the locus under investigation. Additionally, in patient HER2+ 6, allelic instability was observed for 3 non-contiguous SNPs out of 6 informative SNPs. The other 3 non-contiguous SNPs showed results indicative of a stable *HER2/TOP2A* locus. These observations may be the result of a combination of relatively small genetic aberrations each affecting part of the locus.

In the TN group, 75 % of the tumor samples (12/16) showed results indicative of allelic instability of the *HER2/TOP2A* locus. This is in line with previous findings where the complex genomic profiles, with numerous gains and losses, of these tumors were classified as “sawtooth patterns” [23–28]. Nineteen percent of the TN patients (3/16) yielded results that were representative of a stable *HER2/TOP2A* locus. In one sample (patient TN8) the only 2 informative SNPs were found to be equivocal, making the overall result with regard to the stability of the *HER2/TOP2A* locus equivocal.

Within the ERPR+ group, 38 % of the tumor samples (5/13) yielded results representative of allelic instability of the *HER2/TOP2A* locus, whilst 62 % (8/13) did not. Patient ERPR+ 7 showed discordant results in SNP 3, which was therefore concluded to be equivocal. Although the biological and/or technical rationale behind this observation remains unclear, we believe that it is justified to conclude that this sample harbors *HER2/TOP2A* allelic instability due the data of the remaining 5 informative SNPs. Beside the HER2 normal tissue, patient ERPR+ 11 also harbored HER2 amplified tumor tissue, which was most likely caused by the presence of a second tumor or tumor heterogeneity. The tissue included in this study, which had a stable *HER2/TOP2A* locus, was not HER2 amplified and was ERPR positive. This patient was therefore included in the ERPR+ group.

High chromosome instability, detected using FISH, in breast tumors is of prognostic value with regard to clinical outcome and may be useful in predicting response to AC-based therapy [31], since topoisomerase is targeted by AC. It is of particular interest whether these observations are also true for the allelically imbalanced groups found amongst the TN and the ERPR+ patient groups using the presented SNP based assay. Although the number of samples in our study is limited, the portion of tumor samples that contain *HER2/TOP2A* allelic instability in the TN and ERPR+ group (75 % and 38 %, respectively) roughly corresponded to the AC-based chemotherapy response rates found within these groups (85 % and 47 %, respectively) [37]. A future study analyzing patients with available 5 year clinical follow-up data is needed to support this observation.

Overall, 8 out of 10 tissues where part of the tumor cells harbored polysomy of chromosome 17, which was detected by the chromosome 17 centromeric probe included in the SISH assay, showed SNP results indicative of allelic instability. The SNP observations however may be the cumulative effect of several genetic aberrations including the polysomia. Reflection of monosomy and trisomy of chromosome 17 in the SNP results was less evident. In all tumors where aneuploidy chromosome 17 was observed, heterogeneity was observed with regard to chromosome 17 status as well.

SNP 3 cut-off values were adapted due to its large normal ratio distribution. Employing identically calculated cut-off values for SNP 3 would lead to the unjustified normal/equivocal calling of samples that clearly demonstrate a deviant VIC/FAM delta Rn ratio based on the generated scatterplot (data not shown).

In the histologically normal tissues, 99.5 % (207/208) of the informative SNPs yielded results indicative of a stable *HER2/TOP2A* locus. Since sample input of these tissues was similar to those of the tumor tissues, PCR inhibition and depletion of reagents, which may occur, do not seem to be of major impact in allelic stability vs instability calling. The same is true, after the correction of delta Rn FAM values as described earlier, for variation in DNA concentration. The single anomaly concerning SNP 11 that was found aberrant in a histologically normal sample from the breast tumor group may be caused by a small deletion, point mutation(s) or a small recombination event around the involved SNP. Although sequencing of this SNP locus of this particular patient is of interest, it is beyond the scope of this study. Although the SNP test performed well, some drawbacks may be encountered. When analyzing a heterogeneous tumor tissue, unjustified (im)balance calling may occur. The resolution per SNP is relatively low, which may lead to an inconclusive result when (a large part of) the employed SNPs are homozygous and therefore non-informative. However, this issue is easily overcome by increasing the number of SNPs to be analysed. Determining the allelic instability of a sample with the SNP assay, as described here, costs approximately €50-€75 (US $55–85) per sample. These costs are comparable to those of FISH based allelic instability testing. Additionally, the SNP test is well suited for the detection of copy number variations, but is not able to distinguish between various underlying mechanisms (e.g. gains vs. losses). SNP assays have a limited sensitivity, around 20 % “mutant” DNA extracted from whole blood is detectable in a “wildtype” background [32]. For FFPE tissues, this sensitivity might be even somewhat lower. Therefore, to prevent false negativity with regard to allelic instability, the minimum tumor cell percentage was set at 25 %. This implies that allelic imbalance in minor tumor cell populations may have stayed unnoticed. Unfortunately, further analysis of the assays’ sensitivity was not possible due the lack of quantified DNA affected by allelic instability. However, we do not expect DNA degradation to have a significant impact on the assays’ sensitivity because of the small amplicons targeted (up to 80 bp) in combination with the size of amplifiable DNA fragments yielded by the employed DNA extraction method [38].

To minimize the effect of pipetting inaccuracy and to prevent pipetting errors, manual liquid handling was reduced to a minimum by performing all initial PCR setups using a Hamilton StarLet robot. Any inaccuracies or errors with regard to pipetting could negatively influence the determination of the natural ratio distribution of the histologically normal tissues and ultimately the allelic stability vs. instability calling.

## Conclusions

In summary, the presented SNP methodology is easy to perform when compared to other copy number variation detection techniques such as array based systems targeting SNPs and Comparative Genomic Hybridization and corresponds well to positive SISH results. The real time PCR based SNP test may have an added value in the TN as well as the ERPR+ patient group. Within these groups, 75 % of the TN and 38 % of the ERPR+ tumors showed allelic instability with the SNP based assay. To determine such a possible added value of the presented SNP test a large-scale study should be performed. Ample size TN and ERPR+ patient groups with 5 year clinical follow-up data should be included to assess whether the determination of allelic instability of the *HER2/TOP2A* locus as a marker has clinical relevance and is of any significance for therapeutic decision making.

## Additional files

Additional file 1: Table S1. Patient data and SNP test results from the control group. Patient data and SNP results. The top right represents the *HER2/TOP2A* locus and the schematic positions of the employed SNP markers. The arrows indicate the location of the *HER2* and *TOP2A* genes. The boxes represent all informative SNPs that were considered not affected (green)/affected (red) by allelic instability or equivocal (grey). Homozygous SNPs are not informative, and are therefore shown as a horizontal line.

Additional file 2: Table S2. Patient data, tumor characteristics and IHC, SISH and SNP test results from breast tumor group. Patient data, tumor characteristics and IHC, SISH and SNP test results. L and B represent the use of lymph node or breast histologically normal tissue. IHC = immunohistochemistry, SISH = silver *in situ* hybridization, HER2+ = HER2 overexpression observed based on IHC, TN = triple negative, ERPR+ = estrogen receptor and progesterone receptor positive. Chr17 status = chromosome 17 status with M (monosomy), D (disomy), T (trisomy) and P (polysomy) as possible outcomes supplemented with the number of chromosomes 17 observed per nucleus. Pred. = predominantly and TC% = tumor cell percentage. Failed = SISH repeatedly failed. The top right represents the *HER2/TOP2A* locus and the schematic positions of the employed SNP markers. The arrows indicate the location of the *HER2* and *TOP2A* genes. The boxes represent all informative SNPs that were considered not affected (green)/affected (red) by allelic instability or equivocal (grey). Homozygous SNPs are not informative, and are therefore shown as a horizontal line.
